# West Nile virus in Europe: after action reviews of preparedness and response to the 2018 transmission season in Italy, Slovenia, Serbia and Greece

**DOI:** 10.1186/s12992-020-00568-1

**Published:** 2020-05-18

**Authors:** Flavia Riccardo, Francesco Bolici, Mario Fafangel, Verica Jovanovic, Maja Socan, Petra Klepac, Dragana Plavsa, Milena Vasic, Antonino Bella, Gabriele Diana, Luca Rosi, Patrizio Pezzotti, Xanthi D. Andrianou, Marco Di Luca, Giulietta Venturi, Francesco Maraglino, Danai Pervanidou, Orlando Cenciarelli, Agoritsa Baka, Johanna Young, Tamas Bakonyi, Giovanni Rezza, Jonathan E. Suk

**Affiliations:** 1 Department of Infectious Diseases, National Institute of Health (Istituto Superiore di Sanità, ISS), Rome, Italy; 2 OrgLab, University of Cassino and Southern Lazio, Cassino, Italy; 3 Nacionalni inštitut za javno zdravje, Ljubljana, Slovenia; 4 Institut za Javno Zdravlje Srbije “Dr Milan Jovanović Batut”, Belgrade, Serbia; 5 Italian Ministry of Health, Rome, Italy; 6 Hellenic National Public Health Organization, Athens, Greece; 7 European Centre for Disease Prevention and Control (ECDC), Stockholm, Sweden

**Keywords:** Preparedness, West Nile virus, After action reviews, Epidemics, Outbreaks, Infectious disease, Mosquito-borne disease

## Abstract

**Background:**

After Action Reviews (AAR) with a One Health perspective were performed in Slovenia, Italy, Serbia and Greece following a severe West Nile virus (WNV) transmission season in 2018. A protocol combining traditional techniques and organizational process analysis was developed and then implemented in each country.

**Results:**

In 2018, response to the unusually intense transmission season of WNV in Slovenia, Italy, Serbia and Greece took place through routine response mechanisms. None of the four countries declared a national or subnational emergency. We found a very strong consensus on the strengths identified in responding to this event. All countries indicated the availability of One Health Plans for surveillance and response; very high laboratory diagnostic capacity in the human, veterinary and entomology sectors and strong inter-sectoral collaboration with strong commitment of engaged institutions as critical in the management of the event. Finally, countries implementing One Health surveillance for WNV (in terms of early warning and early activation of prevention measures) consistently reported a positive impact on their activities, in particular when combining mosquito and bird surveillance with surveillance of cases in humans and equids. Recurring priority areas for improvement included: increasing knowledge on vector-control measures, ensuring the sustainability of vector monitoring and surveillance, and improving capacity to manage media pressure.

**Conclusions:**

The AARs presented here demonstrate the benefit of cross-sectoral and cross-disciplinary approaches to preparedness for West Nile virus outbreaks in Europe. In the coming years, priorities include fostering and strengthening arrangements that: enable coordinated One Health surveillance and response during WNV transmission seasons; ensure adequate laboratory capacities; strengthen risk communication; and fund longer-term research to address the knowledge gaps identified in this study.

## Introduction and background

### Introduction

In 2018, an unusually early and increased transmission of WNV was documented in several European countries [1–6]. This led to the highest number of cases ever recorded to date in some countries (e.g. Italy, Serbia, Greece) and to the novel identification of local human transmission in others (e.g. Slovenia). In total, EU and EU neighbouring countries reported 2083 human cases and 180 deaths due to WNV infection in 2018 [7]. The overall notification rate of locally acquired human cases of WNV infection in the EU/EEA in 2018 was 0.36/100,000 inhabitants, a 7.2-fold increase compared with the previous year [8].

We performed After Action Reviews (AARs) of the WNV 2018 outbreak event in Italy, Greece, Serbia and Slovenia to understand how each health system responded and systematically identify a set of lessons learned (e.g. best practices and improvement opportunities). Our specific operational protocol enabled the implementation of AARs at national level in a short period of time (max 5 days of site visit) and to include both a macro (how the national health response performed as a system) and a micro level perspective (how different actors involved in the national health system acted and collaborated). We structured our analysis to identify common strengths as well as shared areas of improvement. The aim of this paper is to present the findings of these studies.

### Background: the epidemiology of West Nile virus in Europe

West Nile virus (WNV), a mosquito-borne zoonotic flavivirus, has been emerging in Europe with regular reports of human outbreaks since 1996, mainly in South and South-East European countries, and with a progressive geographic expansion of documented viral circulation [9, 10]. The disease has been under European Union/European Economic Area (EU/EEA) surveillance since 2008, showing a seasonal trend with human cases reported typically from early summer to early autumn with a peak in the warmer summer months. EU/EEA notification rates of locally acquired human WNV infections have ranged between 0.00 and 0.09/100,000 inhabitants between 2008 and 2017 [8].

The enzootic cycle of WNV transmission involves mosquito vectors (predominantly *Culex spp.*) and birds as amplifying hosts. Humans, equids and other mammals, acting as dead-end hosts, can be infected through the bite of infected mosquitoes. Rarely, transmission of WNV through blood transfusion, organ transplantation, breastfeeding, and intrauterine means has been documented [11], making this a relevant pathogen for blood and transplant safety.

Genetic lineages 1 and 2 of WNV are associated with human disease with a median estimated incubation period of 2.6 days [12]. WNV lineage 1 was the main lineage circulating in Europe that was associated with human outbreaks until 2004, when WNV lineage 2 was progressively introduced, becoming the main circulating lineage in the region [9].

While most cases of human infections are thought to be asymptomatic, the virus can cause West Nile Fever and around one in 150 infections progresses to West Nile neuro-invasive disease (meningitis, encephalitis or, more rarely, acute flaccid paralysis), a potentially lethal condition affecting prevalently elderly patients. No specific treatment is available against WNV infection in humans or animals, and no vaccine is available for humans. Inactivated and recombinant vaccines for horses are available and in use in Europe [1].

## Methods

We designed an AAR study to enable the participatory involvement of all the stakeholders potentially involved in a WNV outbreak response (including the human health, animal health, medical entomology/vector control and the blood/transplant safety sectors), targeted to a European context (i.e. with reference to existing relevant EU/EEA and European regulatory frameworks) and with a focus on public health preparedness and response from a One Health perspective and on communication and collaboration mechanisms. We designed the protocol to perform an AAR specifically on the 2018 WNV outbreak event, considering as primary methodological references recent European Centre for Disease Prevention and Control (ECDC) [13] and World Health Organization (WHO) [14] guidance documents.

We adopted a mixed method approach, combining traditional AAR techniques (interviews, working groups), with formalized methodologies from management and organizational studies (e.g. process analysis). Namely we used two methodological references: (i) participatory design techniques to enable the active participation of all the actors involved in the national WNV 2018 outbreak event into the common workshops established by the protocol [15–17]; and (ii) process design and analysis (specifically adopting Business Process Modelling Notation (BPMN) [18]) to identify, model and analyse the communication flows and the coordination needs among the different involved actors, and to map complex surveillance and response pathways [19, 20].

**Fig. 1:**
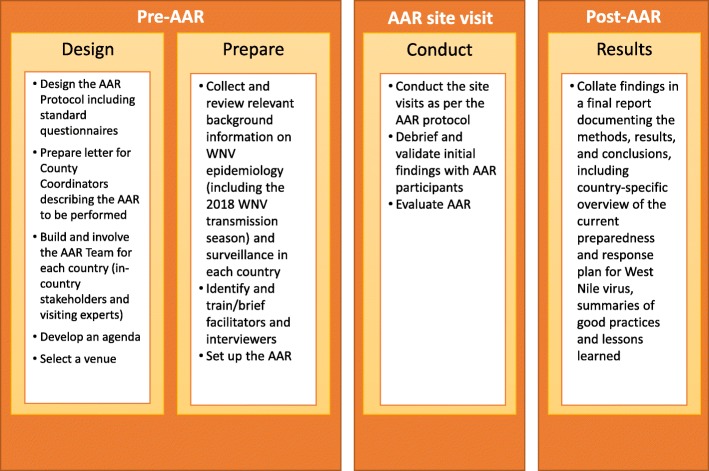
After Action Review Roadmap, adapted from WHO 2019 Guidance for After Action Reviews

**Fig. 2:**
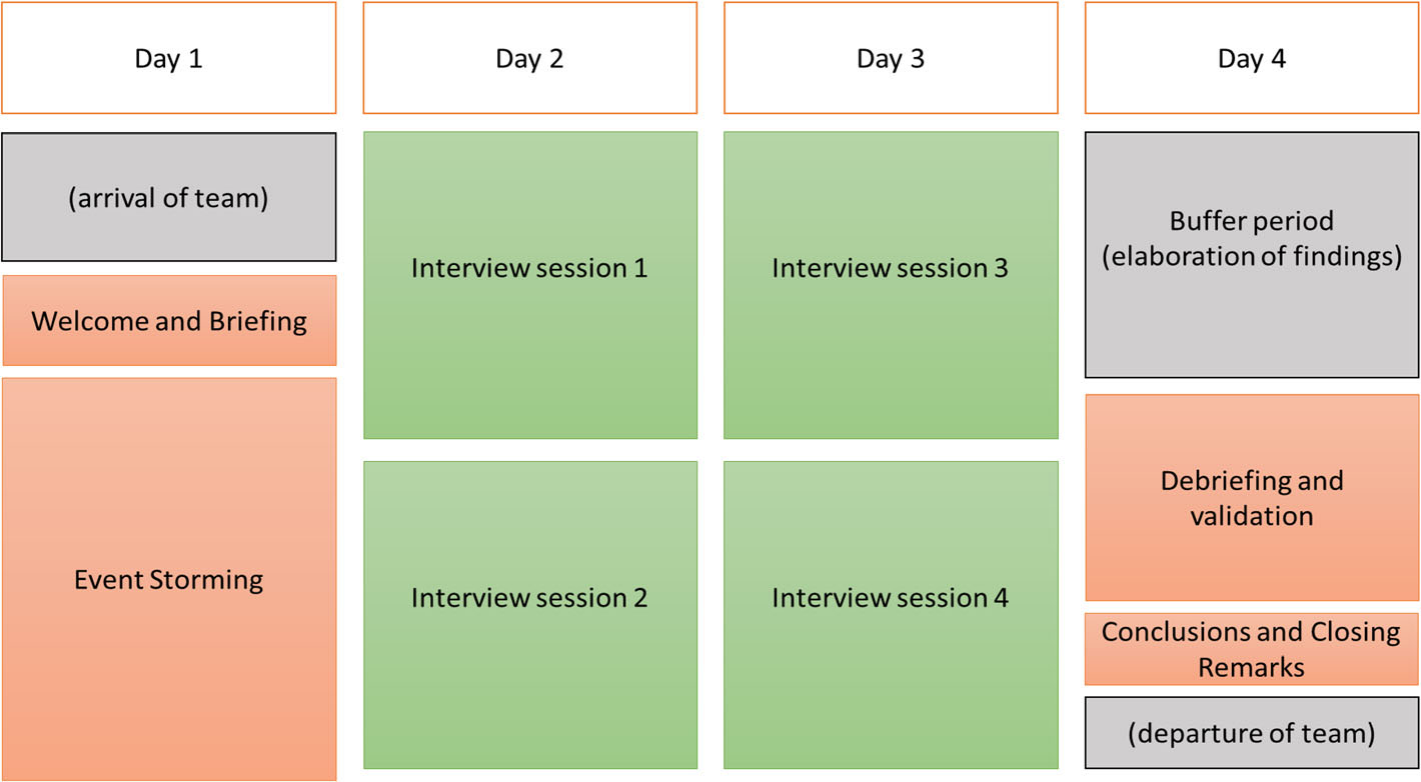
Standard 4-day format for the After Action Review site visits

The AAR was implemented according to pre-defined study steps (Fig. 1) and site visits were planned according to a standard 4-day format (Fig. 2). The methodology was successfully assessed against the ECDC 11-item tool for AAR methodological rigour [16].

We structured the AAR in three different phases (see Table 1): (i) Participants identification; (ii) Data collection & site visits; (iii AAR Quality Check & Feedback.

**Table 1:** AAR structure

Macrophase	Activities
**Participant Identification**	- Recruitment of 2 or 3 team leaders per country involved in the AAR - Recruitment of stakeholders coming from the different national institutions involved in the response to WNV18 season
**Data Collection & Site visits**	- Desk Research/Preparatory activities - Questionnaire design - Plenary workshop for a first high level representation of the process - In-depth interviews with all invited stakeholders to detail process representation - Validation of the designed map with involved stakeholders - Report detailing all strengths and improvement of the processes
**AAR Quality Check & Feedback**	- Survey design to gather impressions and ideas of the proposed WNV methodology - Analysis of the provided answers

### Participant identification

Each participating country was represented by 2–3 team leaders from the national institute of public health/ministry of health. For each AAR, an AAR team was identified combining the hosting country team leaders and international experts. In each hosting country, the officially designated ECDC National Focal Point for Emerging and Vector Borne Diseases was involved either as team leader or as part of the AAR team.

Team leaders within each participating country were in charge for the organization of the AAR they hosted, with the scope to involve all relevant stakeholders at national level, identified through a “stakeholder matrix” tool designed within the protocol (Table 2).

**Table 2:** Stakeholder matrix used to identify stakeholders to involve in the After Action Reviews in Slovenia, Italy, Serbia and Greece

	Human Health	Entomology	Animal Health	Substances of Human Origin (SoHO) Safety
**Surveillance and early warning**	Actors engaged in surveillance of human cases (WNND, fevers; blood donors)	Actors engaged in mosquito surveillance	Actors engaged in surveillance of equids, target/other bird species	
**Policy**	Actors engaged in human health policy (eg MoH)		Actors engaged in animal health policy (eg MoA), eg immunization policies in horses	Actors engaged in SoHO safety policy (if different from actors already engaged)
**Laboratory**	Actors engaged in laboratory testing and confirmation of WNV in humans	Actors engaged in laboratory testing and confirmation of WNV in mosquito pools (if different from actors already engaged)	Actors engaged in laboratory testing and confirmation of WNV in animals	
**Clinical care**	Actors engaged in patient care (eg hospitals)		Actors engaged in animal care (eg horse and wildlife clinics)	
**Vector control**	Actors engaged in vector control related activities and management of alerts	Actors engaged in vector control related activities and management of alerts	Actors engaged in vector control related activities and management of alerts	
**SoHO Safety Measures**				Actors engaged in guiding and implementing SoHO safety measures (screening/deferrals/follow-ups for transplants …)
**Communication**	Actors engaged in communicating with health care providers/ general public	Actors engaged in communicating with general public	Actors engaged in communicating with veterinarians/ general public	Actors engaged in communicating with medical specialists/ general public
**Other relevant**				

All team leaders were encouraged to invite participants from institutions working at national level in the areas identified in the stakeholder matrix. In large countries, where some activities were at least partially decentralized, also stakeholders at sub-national level (regional, provincial, municipal) were involved, to capture a more accurate and context-specific national overview of preparedness and response actions. However, it is worth noting that it was beyond the AAR scope to map each sub-national surveillance and response pathway.

### Data Collection & Site visits

Following a preparatory desk review, each country team leader provided the AAR teams with background information by compiling a standard dedicated questionnaire and providing any relevant documents. This material was collated in an “AAR Country Portfolio” that was distributed to the AAR teams ahead of each AAR visit in order to allow international experts to familiarize in advance with key concepts in WNV epidemiology, preparedness, surveillance and response in each country context.

The AARs in Slovenia, Italy and Serbia were based upon organizational system mapping methodologies and BPMN real-time mapping, while the AAR team in Greece adapted the protocol by extending the visit by 1 day and by not implementing BPMN mapping in real-time. Instead, processes were retrospectively mapped and validated using flow charts.

In all countries, on the first day of the AAR visit, all invited stakeholders were involved in a plenary workshop designed to support their direct participation in system analysis and activities examination. The workshop activities were designed combining different participatory techniques, e.g. an evolution of the event storming [21], to recall and jointly validate the WNV 2018 outbreak event and to agree on the main areas of concerns and prioritise them (these areas of improvement were formalized with particular attention). Discussions naturally led to an exchange of different viewpoints about the transmission season and allowed the building of a consensus driven reconstruction of the event, of the actions that took place and of the difficulties encountered.

In the subsequent days, in-depth interview sessions, based upon a standard semi-structured questionnaire, were conducted with experts from human public health, animal health, medical entomology/vector control and blood and transplant safety. During these interviews, aspects related to preparedness planning, surveillance and response were discussed. In Slovenia, Italy and Serbia, organizational processes were mapped in real-time using BPMN to visually represent communication flows and interdependencies among relevant institutions and actors. The function of the interview leader, note-taker and BPMN mapper were defined ahead of each interview. Both digital and hard copies of the standard questionnaire were available for note taking. Interviews enabled a more in depth discussion within each sector on: preparedness plans in place, what was supposed to happen as per the existing plans during a WNV outbreak and what happened both in terms of surveillance/early detection and response. The repetition of topics across interviews allowed us to triangulate findings and to acquire more detailed feedback on what worked and what could be improved in the short and longer term.

At the end of each day of the site visit, after the interviews, each AAR team met for a debriefing to share notes, discuss content, build a shared understanding of the data collected to that point and identify recurring themes and patterns in its explicit content. BPMN diagrams depicting organizational processes relevant to WNV surveillance and response, were discussed, verified and corrected on the basis of the team’s collective understanding.

Before the last day of the AAR visit, BPMN diagrams, when available, were fully drafted and the main themes that had emerged from the event storming workshop and interviews were identified and summarized to highlight strengths, areas of concerns and what could be changed in the short or longer term. The AAR visit ended with a plenary debriefing workshop in which the findings and organizational processes were discussed and validated with the participating stakeholders.

A report with a synthesis of the AAR results was finalized and shared with the team leaders and AAR teams in June 2019.

### AAR Quality Check & Feedback

**Fig. 3:**
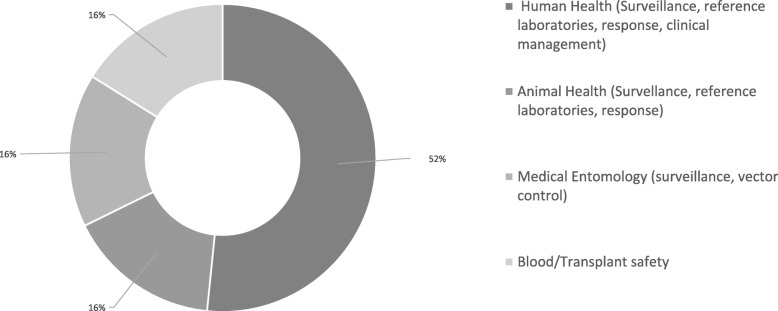
Respondents to the AAR evaluation survey by Sector (n=31/55), all countries

Following the AAR visits, team leaders in each country were asked to evaluate their experience by sending a very short online questionnaire to each institution/authority engaged in the AAR. Frequency distributions of responses by sector were elaborated on the basis of the data provided. A total of fifty-five institutions in Slovenia, Italy, Serbia and Greece, representing all engaged sectors, were invited to respond to the evaluation survey. As shown in Fig. 3, 31 institutions provided a response with an overall response rate of 56% (ranging between 40 and 83% by country). In all countries, respondents recognized that conducting the WNV 2018 outbreak event AAR produced added value for a better understanding and improvement of the system. The majority also agreed that the AARs met the objective of enabling:

- a structured review of response activities to WNV in 2018,
- an exchange of ideas and an in-depth analysis of what happened,
- the identification of current and emerging preparedness gaps,
- good practices and lessons learned and of actions to improve response to the next event

According to responding stakeholders, points of strength of this study have been the ability to:

- engage multiple actors across sectors highlighting individual roles;
- foster in depth discussion, exchange of information and joint practices across disciplines, and eliminate gaps;
- design and discuss in depth existing processes to increase preparedness for the next season;
- conduct a critical review and assessment of any actions and compare different experiences;
- focus on communication processes and coordination.

Respondents recommended that AARs should be conducted with a planned frequency, also targeting sub-national and local levels, trying to limit the number of plenary moments in the site visits (as not all stakeholders could attend the final de-briefing in all countries). Better defining mechanisms to ensure timely feedback on AAR results at all levels, increasing the focus on longer term actions to improve preparedness and response capacity and following-up on identified open questions regarding WNV preparedness and response, were suggested as areas of improvement ahead of future AAR implementation on this topic.

## Results

### Stakeholders involved in the AARs

Between April and May 2019, four AARs were conducted in Slovenia, Italy, Serbia and Greece. In the first three countries, the AAR teams were composed of public health experts from the Italian Institute of Health (Istituto Superiore di Sanità), which coordinated this activity, of team leaders of the hosting countries, of ECDC/WHO experts and of experts in Organization Studies from the Faculty of Economics of the University of Cassino and Southern Lazio. A team of ECDC experts with team leaders of the hosting country led the AAR in Greece.

In all four countries, invited participants were delegates from institutions that worked within all the sectors and for all the activity domains included in the Stakeholder Matrix of the AAR protocol, with the desired representation at national and, where relevant, sub-national level (Tables 3, 4, 5 and 6).

**Table 3:** AAR in Slovenia: stakeholder matrix with participating institutions

	Human Health	Entomology	Animal Health	SoHO Safety
**Surveillance and early warning**	NIJZ	UP; IMI; NLZOH	NVI; PMS; UVHVVR	ZTM; Slovenija-transplant
**Policy**	MZ	MOP	UVHVVR	MZ; JAZMP
**Laboratory**	IMI; NLZOH	IMI; NLZOH	NVI	ZTM
**Clinical care**	KIBVS		VK	
**Vector control**	NIJZ; URSK	NLZOH	UVHVVR	
**SoHO Safety Measures**				ZTM; Slovenija-transplant
**Communication**	NIJZ			

Acronyms/abbreviations of the institutions involved: *IMI* Institute of Microbiology and Immunology, Faculty of Medicine, University of Ljubljana, *JAZMP* Agency for Medicinal Products and Medical Devices of the Republic of Slovenia, *KIBVS* Department of infectious diseases, University Medical Centre Ljubljana, *MOP* Climate Change Section, Ministry of the Environment and Spatial Planning, *MZ* Public Health Directorate, Ministry of Health of the Republic of Slovenia, *NIJZ* National institute of Public Health, *NLZOH* National Laboratory of Health, Environment and Food, *NVI* National Veterinary Institute, Veterinary Faculty, University of Ljubljana, *PMS* Slovenian Museum of Natural History, *Slovenija-transplant* Institute for transplantation of Organs and Tissues of the Republic of Slovenia, *UP* Faculty of mathematics, natural sciences and information technologies, University of Primorska, *URSK* Chemicals Office, Ministry of Health of the Republic of Slovenia, *UVHVVR* Administration of the Republic of Slovenia for Food Safety, Veterinary Sector and Plant Protection, Ministry of Agriculture, Forestry and Food, *VK* Veterinary clinics, Veterinary Faculty, University of Ljubljana, *ZTM* Blood Transfusion Centre of Slovenia.

**Table 4:** AAR in Italy: stakeholder matrix with participating institutions

	Human Health	Entomology	Animal Health	SoHO Safety
**Surveillance and early warning**	ISS-DMI -epidemiology; Region Emilia Romagna; Region Veneto; Region Friuli Venezia Giulia (FVG); Local Health Units FVG	ISS-DMI- entomology; Local Health Units FVG; IZS-AM; IZS-LER; IZS-Ve	IZS-AM; IZS-LER; IZS-Ve	ISS-CNS; ISS-CNT; CRS-FVG
**Policy**	MoH– DG Prev; Regional Health Authority Emilia Romagna Regional Health Authority Veneto; Regional Health Authority Friuli Venezia Giulia; Municipality of Cividale del Friuli	Ministry of Health – DG SAF; Municipality of Cividale del Friuli	Ministry of Health – DG SAF; Municipality of Cividale del Friuli	ISS-CNS; ISS-CNT; CRS-FVG
**Laboratory**	ISS-DMI –NRL; Regional reference laboratory (FVG)	IZS-AM; IZS-LER; IZS-Ve	IZS-AM; IZS-LER; IZS-Ve	ISS-CNS; ISS-CNT; CRS-FVG
**Clinical care**	Infectious Disease Units within Hospitals (S.S Malattie Infettive AAS 5 “Friuli Occidentale”; Azienda Sanitaria Universitaria Integrata di Udine)			
**Vector control**	Municipality of Cividale del Friuli			
**SoHO Safety Measures**				ISS-CNS; ISS-CNT; CRS-FVG
**Communication**	Municipality of Cividale del Friuli; Local Health Units FVG			

Acronyms/abbreviations of the institutions involved: *ISS* National Centre for Health, Italy, *ISS-DMI entomology* ISS Department of Infectious Diseases – medical entomology unit, *ISS DMI – NRL* ISS Department of Infectious Diseases National Reference Laboratory for Arboviruses, *ISS-DMI –epidemiology* ISS Department of Infectious Diseases – epidemiology unit, *ISS-CNS* ISS National Centre for Blood Safety, *ISS: CNT* ISS National Centre for Transplant Safety, *CRS-FVG* Regional Centre for Blood Safety in Friuli Venezia Giulia, *FVG* Friuli Venezia Giulia, *IZS* Veterinary Institute, Italy, *IZSAM* Istituto Zooprofilattico Sperimentale of Abruzzo and Molise, *IZSLER* IZS of Lombardia and Emilia Romagna, *IZSVe* IZS of the Venezie, *ASL* Local Health Unit, *MoH– DG Prev* Ministry of Health Directorate General for Prevention, *MoH– DG SAF* Ministry of Health Directorate General for Animal Health

**Table 5:** AAR in Serbia: stakeholder matrix with participating institutions

	Human Health	Entomology	Animal Health	SoHO Safety
**Surveillance and early warning**	IPH Serbia; IPH Vojvodina; IPH Belgrade IVVS; BTI Serbia	MoAWMF (VD); IBME; SVI; VSI; FoA	MoA; SVI; VSI	BTIS
**Policy**	MoH; MoAWMF	MoH; MoAWMF; SUEPV; SEPB;	MoAWMF	BTIS
**Laboratory**	IVVS Torlak	IBME; SVI; VSI; FoA	SVI; VSI	BTIS
**Clinical care**	CITD			
**Vector control**	PCA; IBME	MoAWMF (VD); IBME; SVI; VSI		
**SoHO Safety Measures**				BTIS
**Communication**	MoH; IPH Serbia; District IPHs	MoAWMF (VD); IBME	MoAWMF (VD)	SEPB

Acronyms/abbreviations of the institutions involved: *MoH* Ministry of Health, *MoAWMF (VD)* Ministry of Agriculture, Water Management and Forestry, Veterinary Directorate; IPHS: Institute of Public Health of Serbia “Dr. Milan Jovanovic Batut”, *IPHV* Institute of Public Health of Vojvodina, *IPHB* Institute of Public Health of Belgrade, *IVVS* Institute for virology, vaccines and sera “Torlak”, *CITD* Clinic for infectious and tropical diseases, Belgrade, *BTIS* Blood transfusion institute of Serbia, *IBME* Institute for Biocides and Medical Ecology, *SUEPV* Secretariat for Urbanism and Environmental Protection of the province of Vojvodina, *SEPB* Secretariat for Environmental Protection of the City of Belgrade, *SVI* Scientific Veterinary Institute “Novi Sad”, *VSI* Veterinary Specialist Institute “Kraljevo”, *FoA* Laboratory for medical and veterinary Entomology of the Faculty of Agriculture, University of Novi Sad, *BM* Becej Municipality, Department responsible for organizing mosquito control in the municipality, *PCA* Pest control agency.

**Table 6:** AAR in Greece: stakeholder matrix with participating institutions

	Human Health	Entomology	Animal Health	SoHO Safety
**Surveillance and early warning**	NPHO-VBDO; CHVC; HNBTC; HNTO; WGDAA	RCM-PH (PC); RUWA-EH (PC); RA-PH; NSPH; NPHO-VBDO; BPI	MoRD&F-VS	CHVC; HNBTC; HNTO; WGDAA
**Policy**	MoH-PH	MoH-PH; RCM-PH; RUWA-EH; RA-PH; BPI	MoRD&F-VS	HNBTC; CHVC; HNTO
**Laboratory**	NRCA	NSPH	MoRD&F-VS	HNBTC; NRCA
**Clinical care**	Infectious Diseases Experts- NPHO Consultants			
**Vector control**	RCM-PH (PC); RUWA-EH (PC); RA-PH (PC)	RCM-PH (PC); RUWA-EH (PC); RA-PH (PC)	RCM-PH (PC); RUWA-EH (PC); RA-PH (PC)	
**SoHO Safety Measures**	HNBTC; CHVC; HNTO			HNBTC; CHVC; HNTO
**Communication**	NPHO-VBDO; NPHO- PCO; MoH-PH; RCM-PH; RUWA-EH; RA-PH	NPHO-VBDO; RCM-PH; RUWA-EH; RA-PH	MoRD&F-VS	HNBTC; CHVC; HNTO

Acronyms/abbreviations of the institutions involved: *MoH-PH* Ministry of Health [includes General Secretariat for Public Health, General Directorate of Public Health and Quality of Life, and a multi-sectorial National Committee for the Management and Prevention of Tropical Diseases], *NPHO* Hellenic National Public Health Organization (MoH), *NPHO-VBDO* NPHO-Department of Epidemiological Surveillance and Intervention, Vector-borne Diseases Office, *NRCA* National Reference Center for Arboviruses and Haemorrhagic Fever viruses, Medical School, Aristotle University of Thessaloniki, *RCM-PH* Region of Central Macedonia- General Directorate of Public Health, *RUWA-EH* Regional Unit of West Attica, Region of Attica- Directorate of Environmental Hygiene and Sanitary Control, *RA-PH* Region of Attica- General Directorate of Public Health, *PC* Private companies- contractors of the vector control programmes of the Region of Central Macedonia (“Ecodevelopment SA”) and the Regional Unit of West Attica (“Inseko LP & Bioefarmoges Eleftheriou & Co LP”), *HNBTC* Hellenic National Blood Transfusion Centre (Laboratory screening testing of blood donations is performed in the HNBTC in Athens and in the AHEPA University Hospital Blood Centre in Thessaloniki), *CHVC* Coordinating Haemovigilance Centre of the Hellenic NPHO, *HNTO* Hellenic National Transplant Organization, *NPHO-PCO* NPHO- Press & Communication Office, *NSPH* National School of Public Health, *BPI* Benaki Phytopathological Institution, *MoRD&F* Ministry of Rural Development & Food (the implementation of the animal surveillance programme is performed by the Regional Veterinary Authorities), *MoRD&F-VS* MoRD&F’s Directorate General of Veteninary Services, *WGDAA* Working Group for the designation of affected areas from vector-borne diseases- under the National Committee for the Management and Prevention of Tropical Diseases (MoH).

### Main findings

In 2018, response to the unusual transmission season of WNV in Slovenia, Italy, Serbia and Greece took place through routine response mechanisms. None of the four countries declared a national or sub-national emergency notwithstanding the unusually high overall number of human cases and deaths compared with previous years.

### Strengths and organizational processes

We found a very strong consensus in terms of the strengths identified in all four countries during the response to the 2018 WNV transmission season. Inter-sectorial preparedness and planning was well established in all countries with availability of One Health Plans for surveillance and response that were consistently described as supportive to managing the event.

Very high diagnostic capacity for WNV, also considering existing diagnostic difficulties due to cross-reactions among flaviviruses, were consistently identified as an important point of strength in the human, veterinary and entomology sectors. Capacity building and/or maintenance in order to adequately and promptly detect infection in humans, horses, birds and mosquitoes was consistently highlighted as crucial in ensuring timeliness and completeness of surveillance.

All countries also described working in a context of strong inter-sectoral collaboration (both at formal and informal level) with strong commitment of the involved institutions. This reportedly favoured the establishment of consolidated mechanisms for inter-sectoral rapid exchange of information and consensus on triggers for action.

Timeliness and integration of surveillance at the human-animal interface was identified as a point of strength in all four countries, while activities around entomological surveillance appeared to be more consolidated and integrated in countries where transmission had been documented for a longer time. The added value of One Health surveillance for WNV has been consistently reported by countries implementing it, in terms of early warning and early activation of prevention measures, in particular, when combining mosquito and bird surveillance with surveillance of cases in humans/equids, due to the fact that on average virus detection by PCR occurs earlier in those species (Table 7).

**Table 7:** Recurring strengths, lessons learned, ongoing actions and related strategic lines, WNV AARs in Slovenia, Italy, Greece and Serbia, April–May 2019*

Recurring Strengths	Lesson Learned	Ongoing actions	Derived Strategic Line
1. *Intersectoral preparedness and planning, with availability of One Health Plans for surveillance and response has been consistently described as supportive to the management of the WNV transmission season.*	A formal legal framework and mandate sustaining the implementation of One Health activities including, but not limited to, surveillance is recurrently recognized as a strength.	In reaction to the WNV transmission season 2018 Italy, Slovenia and Serbia increased formalization of existing committees (through the formal nomination of higher level/broader groups). Italy engaged in the production of a longer term and higher-level preparedness and response plan. Greece has an established national inter-sectoral committee (under the MoH) and a multi-sectoral working group which provides criteria for the designation of affected areas.	Invest in, and if possible strengthen the formal inter-sectoral framework that is supportive to the implementation of coordinated One Health surveillance and inter-sectoral response during the WNV transmission seasons
2. *Inter-sectoral collaboration*	Established (formal and informal) mechanisms of collaboration and communication (including rapid sharing of surveillance data) across the human public health, animal health, medical entomology, and Substances of Human Origin (SoHO) safety sectors were described in all the countries. Technical experts in the different sectors are described consistently as strongly interconnected with a clear understanding of respective roles and responsibilities. All countries identified the rapid detection and investigation of human cases of West Nile Neuroinvasive Disease (WNND) infection through enhanced human surveillance and information sharing as a point of strength. Activities around entomological surveillance and vector control appear more consolidated in countries where transmission has been documented for a long time.	A project has been approved in Slovenia to pilot mosquito surveillance of WNV The Region of Vojvodina in Serbia will design and pilot the implementation of a One Health surveillance platform. This project, funded in 2019, will be implemented in 2020.	Where feasible, establish mosquito and bird surveillance of WNV integrated with the surveillance of cases in human and, if possible, equids. An added value has been consistently reported by countries implementing One Health surveillance for WNV in terms of early warning and early activation of prevention measures, in particular when combining mosquito and bird surveillance with surveillance in cases in humans due to the fact that on average virus detection by PCR occurs earlier in those species.
3. *Enhanced surveillance timeliness and integration at the human-animal interface*			
4. *Strong commitment of engaged institutions*			
5. *Diagnostic capacity (human and veterinary health)*	Capacity within national (and in some countries regional) laboratories for the detection of WNV infection in humans, birds, horses and mosquitoes was highlighted in all countries.		Strengthen technical capacity and the network of reference laboratories for WNV wherever needed. Capacity building or maintenance to detect infection in humans, mosquitoes, birds and horses was consistently highlighted as crucial in ensuring timeliness and completeness of surveillance.

* The identified points of strength were commonly identified by all the four countries that conducted the AAR on the WNV 2018 transmission season.

**Fig. 4:**
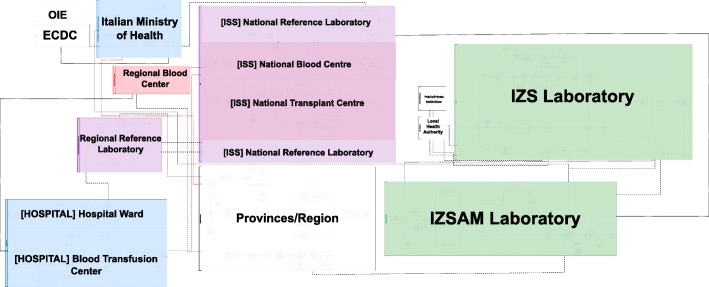
Visual representation (BPMN) of the macro-level organizational systems for WNV preparedness, surveillance and response in 2018, (from top to bottom) Slovenia, Italy and Serbia Legend: IMI: Inštitut za mikrobiologijo in imunologijo, Medicinska fakulteta, Univerza v Ljubljani=Institute of Microbiology and Immunology, Faculty of Medicine, University of Ljubljana; NIJZ: Nacionalni inštitut za javno zdravje=National institute of Public Health of Slovenia; Biocide (Slovenia): Chemicals Office – use of biocidal products in the environment, Environment and Food– disinsection, virology and surveillance; OiE: World Organization for Animal Health; ECDC: European Centre for Disease Prevention and Control; ISS: Istituto Superiore di Sanità – National Centre for Health, Italy; IZS: Istituto Zooprofilattico Sperimentale – Veterinary Institute, Italy; IZSAM: Istituto Zooprofilattico Sperimentale dell’Abruzzo e del Molise; ASL: Local Health Unit, Italy; MoAWMF (VD): Ministry of Agriculture, Water Management and Forestry, Veterinary Directorate; IPH-S: Institute of Public Health - Serbia "Dr. Milan Jovanovic Batut"; HCC: Clinical Centre and Hospitals; Biocide (Serbia): Institute for biocides, Serbia

The organization for WNV surveillance and response activities across the involved sectors varied substantially between the four countries in complexity and centralisation that depended on the size and organizational structure of each health system. BPMN mapping in Italy, Slovenia and Serbia (Fig. 4) highlighted in all the countries high levels of information exchange between sectors, both through the use of formal channels (e.g. online systems and databases, reports, medical records) and – especially when time was essential – informal (e.g. direct calls and personal emails). Another similarity among those national systems is the clear need to have all the inter-sectoral information related to the WNV surveillance and response activities, easy to access and to read in real-time. The organizational analysis clearly highlights that the system is highly decentralized in Italy, while in Serbia and Slovenia the processes are managed with a higher level of centralization, partially due to territorial and size differences. Another difference is in the level of formalization of some support activities, ranging from supply procurement to media management. In some countries those activities are more standardized while in others they are more influenced by the emerging initiatives of different actors, often stepping up to fill an organizational gap.

### Recurring specific problems

We also found areas identified by two or more countries as a high priority to address in order to improve WNV preparedness and response capacity in the future. Increasing knowledge to solve uncertainties around effectiveness and impact of vector-control measures was identified as a priority in three countries. Specifically, lack of strong evidence on the impact of the use of biocides in preventing transmission of WNV infection to humans was mentioned as a critical factor in Italy, where most human cases are documented in semi-urban and rural contexts, in Greece, where human cases are documented in both urban and rural contexts, and in Slovenia, where the possibility of licensing insecticides for outdoor use is being discussed. Ensuring the sustainability of vector monitoring (e.g. to generate nationally standardised maps of distribution of potential WNV vectors) and surveillance activities was identified as critical in Serbia and Slovenia. Specifically, the need to better define the legal framework, mandate and funding for this activity was described as crucial in ensuring the establishment or the sustainability of existing vector monitoring/surveillance programmes. Serbia and Greece highlighted how the complexity and lack of flexibility of procurement processes are major issues also for the planning and implementation of vector control measures. Finally, the exceptional transmission season in 2018 led to an increase in media pressure in all the countries that underwent the AARs. This pressure was described as very high at the national level in Greece, Serbia and Slovenia while in Italy this pressure was more evident at the sub-national level. Improving capacities to manage media pressure during WNV transmission peaks was therefore identified by all countries as an aspect to address in the short/longer term to improve overall response capacity (Table 8).

**Table 8:** Recurring “Specific Problems”, lessons learned, ongoing actions and related strategic lines, WNV AARs in Slovenia, Italy, Greece and Serbia, April–May 2019*

Recurring “Specific Problems”	Lesson Learned	Ongoing actions	Derived Strategic Line
1. *Lack of strong evidence on the impact of the use of biocides in preventing transmission of WNV infection to humans was mentioned as a critical factor in Italy, where most human cases are documented in semi-urban and rural contexts, in Greece, where human cases are documented in both urban and rural contexts, and in Slovenia (where the possibility of licensing insecticides for out-door use is being discussed).*	Persisting “unknowns” related to WNV such as the role of certain bird species in WNV transmission and ecology, the impact of vector control on human transmission and on the development of resistance to insecticides were consistently described in interviews as elements of fragility undermining the implementation of solid preparedness and response plans against this disease.		The development of a research agenda for the “known unknowns”, not only at national but also at international level, could also be advocated for in the longer term. Where feasible, with a long term prospect, research activities at national level could include the assessment/monitoring of vector control effectiveness/efficacy and surveillance of resistance to insecticides (suggested by Italy and Greece) and the in depth mapping and continuous monitoring of breeding sites (suggested by Serbia).
2. *Sustainability of vector monitoring and surveillance*	Legal framework, mandate and funding allocations were described as crucial in ensuring the establishment and the sustainability of existing vector monitoring/surveillance programmes in particular in Slovenia and in Serbia.	A project has been approved in Slovenia to pilot mosquito surveillance of WNV In Greece, active vector surveillance at the national level is being organised for 2019 (as occurred in some previous years)	Where feasible, advocate for the relevance of vector monitoring (e.g. to generate nationally standardised maps of distribution of potential WNV vectors) and surveillance activities in improving WNV preparedness and response. Promotion of harmonized legislation with reference to entomological surveillance mandate and budget could be a strategy to clarify roles and responsibilities.
3. *Lack of flexibility of procurement processes*	Serbia and Greece highlighted the complexity and lack of flexibility of procurement processes as a major issue for example in aspects related to vector control.	Serbia has established a high-level committee to improve the implementation of vector control activities.	In the framework of national legislation, foster the adoption of procurement services and procedures that can facilitate the prompt implementation of activities for WNV surveillance and response, including procurement of biocides and appropriate and timely implementation of vector control activities.
4. *Media pressure*	Due to the exceptional transmission season, 2018 led to an increase in media pressure in all the countries that underwent the AARs. This pressure was described as very high at the national level in Greece, Serbia and Slovenia while in Italy this pressure was more evident at the sub-national level.		Strengthen skills and capacity of public health staff in communication and media management by encouraging training targeting One Health professionals and, where relevant, produce WNV communication standard operating procedures across sectors.

* The points in this table were mentioned by at least two of the four countries that conducted the AAR on the WNV 2018 transmission season. The lessons learned column provides details on the specific context and on the countries each point refers to.

The AARs consistently identified lessons learned for each recurring strength and specific problem (Table 7, Table 8). Shorter- and longer-term actions to improve preparedness were highlighted within each country. Some activities towards these actions were already being implemented at the time the AAR visits were performed.

## Discussion

An AAR on the 2018 WNV transmission season in Europe was successfully implemented in three very different country contexts (i.e. Slovenia, Italy and Serbia) without requiring any adjustment in the content, structure or tools in the protocol. The structure of the protocol proved flexible enough to enable a fourth study in Greece using simplified retrospective mapping with comprehensive results. In all countries, the AAR successfully brought stakeholders together to discuss the public health response to the WNV 2018 outbreak event. The opening participatory workshop involved all participating institutions and created a solid and shared foundation for the in-depth interviews of the following days. It helped to address collective knowledge gaps, align perspectives across sectors, and in some cases even to identify immediate measures that could be undertaken to improve the response to the 2019 transmission season. Organisational system mapping, where performed, highlighted clearly defined communication flows in the different WNV preparedness and response systems. We consistently highlighted high levels of information exchange between sectors and a strong effort to produce easy to access and to read near real-time inter-sectorial information related to the WNV surveillance and response activities. These points are in line with the coordination needs of a complex and diversified network of different organizations, which intensify their collaboration for specific events and have to establish a mutual cooperative behaviour [22–24].

The four countries described in this study have very diverse experiences in relation to WNV (endemic countries with hundreds of human cases vs newly affected countries with few human cases reported in 2018) and, also, very diverse in terms of geographical extension, population size and public health system organization. All four countries had preparedness plans and multi-sectorial surveillance and response systems in place prior to the 2018 outbreak and shared similar strengths, mainly linked to cross-sectoral coordination between actors. This suggests that the investment in preparedness to face the WNV 2018 outbreak event was recognized, on hindsight, as an asset. Conversely, the problems identified were more context specific. Nonetheless, both recurring strengths as well as recurring specific problems were identified. As concerns the latter (recall Table 8), these are very frequently matters that could be addressed at the national and sub-national level if appropriate levels of strategic attention can be harnessed, which is inherently challenging. Activities that can facilitate this include assessing the added-value of critical activities, such as sustained mosquito surveillance programs, in order to ensure appropriate funding. Planning for specific activities, such as media training and operational arrangements to reduce strain on front-line public health workforce during outbreaks, can help to alleviate the strain caused by increased media attention during crises. On the other side, administrative arrangements to streamline or at least pre-consider procurement of biocides and other relevant materials required during outbreaks of WNV, often involve a multiplicity of actors and administrative levels. In some instances, countries may first need to weigh the efficacy of various biocides with their environmental impacts. If this aspect can be prioritized in the aftermath of the 2018 outbreak, it would call for a long-term planning and preparedness process that should preferably take place during “peace-time”. Finally, the scientific community may embark upon research to address the key “known unknowns” surrounding WNV, which have included the role of certain bird species in WNV transmission and ecology; the impact of vector control on human transmission; and the development of resistance to insecticides. As these issues are relevant for all countries that experience WNV transmission, international research projects might be a feasible path forward.

Meanwhile, it will be important to pay adequate attention to the identified recurring strengths (Table 7) to ensure that these are safeguarded and built upon. Many of the strengths relate to inters-sectoral frameworks that enable a One Health approach to WNV. Mechanisms that continue to foster trust and collaboration between relevant agencies and that enable the prompt an efficient exchange of relevant information, including surveillance data, are likely to remain of particular importance. Finally, efforts should be allocated to ensure that laboratory systems continue to adequately diagnose WNV infection across humans, birds, mosquitos and horses.

### Limitations

The AAR protocol described here was effective in providing fast feedback at the end of each site visit and a rapid summary report to the main actors involved in each AAR. However, as suggested by stakeholders, this process could be refined further to facilitate the production of more in depth and detailed reports within a short time frame for the use of national and sub-national participants. Further, the timeframe for the design and implementation of these AARs did not allow for the development of a follow-up plan to assess the medium- and longer-term impact of the recommendations issued. Follow-up actions after AARs are recommended by WHO and were suggested by participating stakeholders. This aspect could be discussed in more detail and defined with implementing countries when planning to perform AARs in the future.

## Conclusions

The preparedness mechanism of the four European countries that implemented the AAR proved to be strong enough to sustain the impact of the extraordinary 2018 transmission season. The WNV AAR met the objectives defined by both ECDC and WHO for this type of study and was recognized as an added value in implementing countries.

In the context of WNV, this study highlights the importance of a cross-sectoral and cross-disciplinary approach to strengthening preparedness to infectious disease threats. Formal inter-sectoral frameworks that support coordinated One Health surveillance and response – within and across national borders – during WNV transmission seasons, may be increasingly important in this era of climate change and intensive agriculture. Laboratory capacities to ensure timely and accurate confirmation must be safeguarded and strengthened where necessary. Risk communication, especially given the rapid pace of change in social media, requires sustained investment and training across public health domains. Finally, where common knowledge gaps remain, such as on the longer-term “known unknowns” identified in this study, there is value in pursuing multi-country research partnerships, sponsored by regional and/or international bodies.

## Data Availability

Data sharing is not applicable to this article as the study was qualitative and no datasets were generated or analysed during the current study.

## Abbreviations

AAR: After Action Reviews
ECDC: European Centre for Disease Prevention and Control
SoHO: Substances of Human Origin
WHO: World Health Organization
WNND: West Nile Neuro-invasive Disease
WNV: West Nile virus
