# Correction to: West Nile virus in Europe: after action reviews of preparedness and response to the 2018 transmission season in Italy, Slovenia, Serbia and Greece

**DOI:** 10.1186/s12992-020-00580-5

**Published:** 2020-07-02

**Authors:** Flavia Riccardo, Francesco Bolici, Mario Fafangel, Verica Jovanovic, Maja Socan, Petra Klepac, Dragana Plavsa, Milena Vasic, Antonino Bella, Gabriele Diana, Luca Rosi, Patrizio Pezzotti, Xanthi D. Andrianou, Marco Di Luca, Giulietta Venturi, Francesco Maraglino, Danai Pervanidou, Orlando Cenciarelli, Agoritsa Baka, Johanna Young, Tamas Bakonyi, Giovanni Rezza, Jonathan E. Suk

**Affiliations:** 1 Department of Infectious Diseases, National Institute of Health (Istituto Superiore di Sanità, ISS), Rome, Italy; 2 OrgLab, University of Cassino and Southern Lazio, Cassino, Italy; 3 Nacionalni inštitut za javno zdravje, Ljubljana, Slovenia; 4 Institut za Javno Zdravlje Srbije “Dr Milan Jovanović Batut”, Belgrade, Serbia; 5 Italian Ministry of Health, Rome, Italy; 6 Hellenic National Public Health Organization, Athens, Greece; 7 European Centre for Disease Prevention and Control (ECDC), Stockholm, Sweden

**Correction to: Glob Health 16, 47 (2020)**

**https://doi.org/10.1186/s12992-020-00568-1**

Following publication of the original article [1], the authors flagged that the article had published with an incomplete version of Fig. 4; only the Italian part of the figure was detailed.

The original article [1] has since been updated with the complete (corrected) figure.

Please also find the corrected figure in this article for reference (Fig. 4).

**Fig. 4:**
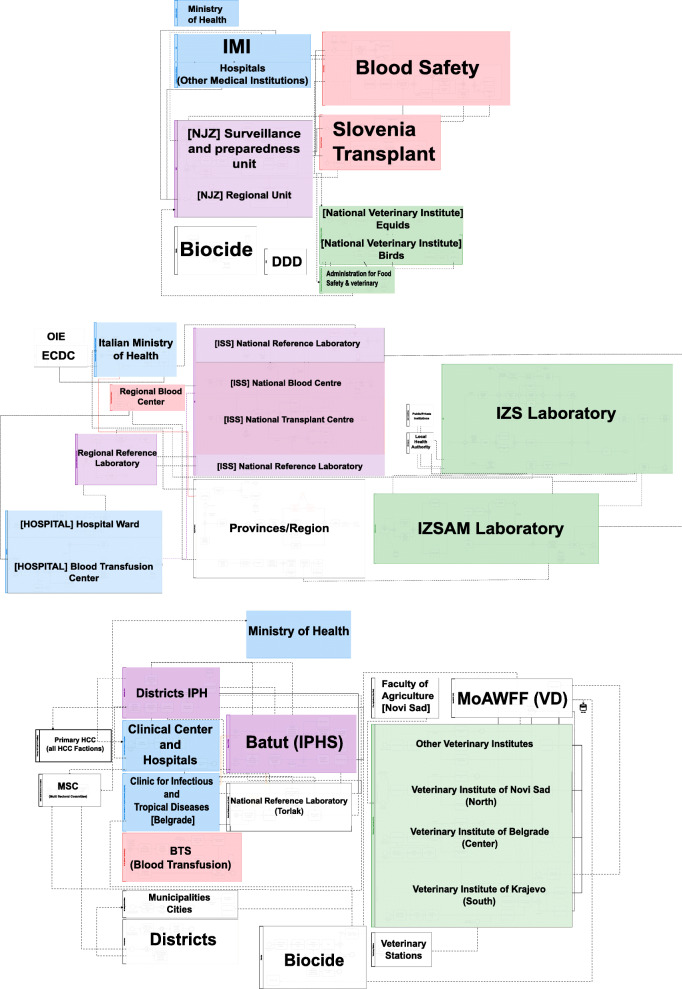
Visual representation (BPMN) of the macro-level organizational systems for WNV preparedness, surveillance and response in 2018, (from top to bottom) Slovenia, Italy and Serbia Legend: IMI: Inštitut za mikrobiologijo in imunologijo, Medicinska fakulteta, Univerza v Ljubljani=Institute of Microbiology and Immunology, Faculty of Medicine, University of Ljubljana; NIJZ: Nacionalni inštitut za javno zdravje=National institute of Public Health of Slovenia; Biocide (Slovenia): Chemicals Office – use of biocidal products in the environment, Environment and Food– disinsection, virology and surveillance; OiE: World Organization for Animal Health; ECDC: European Centre for Disease Prevention and Control; ISS: Istituto Superiore di Sanità – National Centre for Health, Italy; IZS: Istituto Zooprofilattico Sperimentale – Veterinary Institute, Italy; IZSAM: Istituto Zooprofilattico Sperimentale dell’Abruzzo e del Molise; ASL: Local Health Unit, Italy; MoAWMF (VD): Ministry of Agriculture, Water Management and Forestry, Veterinary Directorate; IPH-S: Institute of Public Health - Serbia “Dr. Milan Jovanovic Batut”; HCC: Clinical Centre and Hospitals; Biocide (Serbia): Institute for biocides, Serbia
